# Asthme et obésité: relation et implications thérapeutiques auprès des patients asthmatiques du Service de Pneumologie de Monastir, Tunisie

**DOI:** 10.11604/pamj.2020.36.49.21098

**Published:** 2020-06-01

**Authors:** Saousen Cheikh Mhamed, Ahmed Ben Saad, Asma Migaou, Nesrine Fahem, Naceur Rouatbi, Samah Joobeur

**Affiliations:** 1 Service de Pneumologie et d’Allergologie, Hôpital Universitaire Fattouma Bourguiba, Rue 1^er^ juin, 5000 Monastir, Tunisie

**Keywords:** Asthma, obesity, respiratory functional exploration, control, treatment, Asthme, obésité, exploration fonctionnelle respiratoire, contrôle, traitement

## Abstract

**Introduction:**

L’obésité et l’asthme sont deux maladies chroniques touchant des millions d’individus à travers le monde. La présence d’un lien de causalité est suggérée. L'objectif de notre travail est d'étudier le profil de l’asthmatique obèse et de déterminer la relation entre les différents paramètres de sévérité de l’asthme avec les grades de l’obésité.

**Méthodes:**

Il s'agit d'une étude rétrospective, monocentrique, analytique menée au Service de Pneumologie et d’Allergologie au CHU Fattouma Bourguiba de Monastir portant sur 450 asthmatiques, ayant un indice de masse corporelle (IMC) ≥ 30 kg/m^2^ avec un recul d’au moins 6 mois.

**Résultats:**

L’âge moyen au moment du diagnostic était de 45±12.8 ans. L’IMC moyen était de 34,8±4,2 kg/m^2^. L’asthme était bien contrôlé chez 55,3% des patients. Des critères de sévérité étaient notés dans 37.4% des cas. Selon GINA 2016, 24,2% sont traités par le palier 4. Deux phénotypes de l’asthme associé à l’obésité étaient notés. Le premier phénotype (52,4%) était caractérisé par un asthme à début précoce, associé à une fréquence plus élevée d’allergie, et des manifestations d'atopie. Le deuxième (47,6 %) était caractérisé par un asthme à début tardif, fréquemment associé au sexe féminin et un taux plus élevé de comorbidités et d’hospitalisations. Les obèses de grade II et III avaient un déficit ventilatoire important (CVF: p = 0,002 et VEMS: p = 0,007).

**Conclusion:**

L’obésité est l’un des facteurs clefs impliqués dans le mauvais contrôle de l’asthme. Sa prise en charge, qui n'est pas encore codifiée, doit être multidisciplinaire.

## Abstract (en)

**Introduction:**

Obesity and asthma are two chronic diseases affecting millions of people around the world. A causal relationship has been suggested. The purpose of our study is to examine the profile in obese people with asthma and to determine the relationship between the different severity parameters in asthma and the degree of obesity.

**Methods:**

We conducted a retrospective, monocentric, analytical study involving 450 asthmatics with a body mass index (BMI) ≥ 30 kg/m^2^ having follow up visit at less than 6 months. The study was conducted in the Department of Pneumology and Allergology at the Fattouma Bourguiba Hospital in Monastir.

**Results:**

The average age at diagnosis was 45±12.8 years. Mean BMI was 34.8±4.2 kg/m^2^. Asthma was well-controlled in 55.3% of patients. Severity criteria were reported in 37.4% of cases. According to GINA 2016, 24.2% of patients received treatment at step 4. Two asthma phenotypes associated with obesity were reported. The first phenotype (52.4%) was characterized by early-onset asthma associated with a higher incidence of allergic disease and manifestations of atopy. The second phenotype (47.6%) was characterized by late onset asthma, commonly occurring in female sex as well as a higher rate of comorbidities and hospitalizations. Patients with class II and III obesity had significant ventilatory deficiency (CVF: p = 0.002 and FEV1: p = 0.007).

**Conclusion:**

Obesity is one of the key factors involved in poor asthma control. Its management, which has not yet been codified, should be multidisciplinary.

## Introduction

L’obésité et l’asthme sont deux maladies chroniques touchant des millions d’individus à travers le monde [1, 2]. Ces deux pathologies constituent par leur fréquence croissante un véritable défi pour le clinicien et pour la santé publique [3]. En effet, 39% des adultes âgés de plus de 20 ans à travers le monde sont en surpoids et 13% sont obèses [4]. La prévalence de l’asthme dans le monde est de 1 à 18% [5]. L’obésité est impliquée dans la limitation de la fonction respiratoire [6]. Elle interagit avec plusieurs maladies respiratoires comme le syndrome d’apnée de sommeil et les bronchopneumopathies chroniques obstructives [7]. Actuellement, la présence d’un lien de causalité entre l’obésité et l’asthme est suggérée [8]. L’augmentation de la prévalence et l’incidence des deux pathologies n’est plus considérée comme une simple coïncidence [9]. Il s’agit selon plusieurs études d’une relation dose-réponse [3]. Néanmoins les mécanismes physiologiques de cette association sont encore mal élucidés [3]. Sur le plan clinique, l’asthme de l’obèse est reconnu être résistant au traitement conventionnel avec un retentissement important sur la qualité de vie [10-12]. Il constitue ainsi un phénotype particulier de l’asthme dont la prise en charge n’est pas encore codifiée devant l’absence de données exhaustives sur cette entité clinique. Dans cette étude, nous avons étudié le profil de l’asthmatique obèse afin de déterminer la relation entre les différents paramètres de sévérité de l’asthme avec les grades de l’obésité.

## Méthodes

Il s’agit d’une étude observationnelle, rétrospective, monocentrique, analytique, incluant des patients suivis pour asthme au service de Pneumologie et d’Allergologie à l’Hôpital Universitaire Fattouma Bourguiba de Monastir. Cette étude a été menée sur une période de 17 ans allant de janvier 1999 à décembre 2016. Les sujets inclus dans notre étude sont des sujets âgés de plus de 15 ans suivis pour asthme, et ayant un indice de masse corporelle (IMC) ≥ 30 kg/m^2^ avec un recul d’au moins 6 mois. Le diagnostic de l’asthme a été retenu devant la présence d’une histoire clinique évocatrice (dyspnée sifflante, oppression thoracique…) et devant la positivité des tests de réversibilité aux bronchodilatateurs ou des tests de provocation bronchique non spécifique à la méthacholine. Un test de réversibilité positif a été défini par une augmentation du volume expiré à la première seconde lors d’une expiration forcée (VEMS) d’au moins 200 ml et au moins 12% de la valeur initiale après inhalation d’un bronchodilatateur. Un test de provocation bronchique non spécifique à la méthacholine (à des doses croissantes jusqu’à 3000 µg) a été considéré positif si le VEMS diminue d’au moins 20% par rapport à la valeur initiale. Les sujets pouvant avoir un syndrome de chevauchement asthme BPCO (Asthma COPD Overlap: ACO) étaient exclus de l’étude. Il existe trois grades d'obésité: grade I pour un IMC compris entre 30 et 34,9 kg/m^2^, grade II: 35 et 39,9 kg/m^2^, et grade III: IMC ≥ 40 kg/m^2^.

Les patients ont été évalués sur le plan épidémiologique (l’âge, le sexe, la profession, le niveau socioéconomique, le tabagisme, l'indice de masse corporelle (IMC), présence ou non d’atopie, comorbidités), clinique (sévérité de l’asthme, étiologie), fonctionnelle (exploration fonctionnelle respiratoire), étiologique (tests cutanés allergiques, dosage sériques des IgE spécifiques, examens radiologiques), thérapeutique (paliers thérapeutiques, traitement étiologique) et évolutive (le niveau de contrôle de l’asthme). Dans cette étude, on a utilisé la classification GINA (Global Initiative for Asthma) 2016 pour l’évaluation de la sévérité et le contrôle de l’asthme [13]. Le niveau de contrôle de l’asthme a été évalué à chaque consultation en se basant sur la fréquence des symptômes diurnes et nocturnes, la fréquence des exacerbations, les besoins en bronchodilatateur ainsi que la limitation de l'activité physique. Ainsi, on a défini 3 niveaux de contrôle: asthme bien contrôlé, partiellement contrôlé, et non contrôlé. Les patients ont été ainsi répartis en 2 groupes selon l’âge du début des symptômes. Le groupe 1 (D < 40) est le groupe incluant les patients dont les symptômes ont commencé avant l’âge de 40 ans. Le groupe 2 (D ≥ 40) est le groupe incluant les patients dont les symptômes ont commencé après l’âge de 40 ans. L’analyse statistique a été réalisée à l’aide du logiciel SPSS version 22 (Statistical Package for the Social Sciences). Nous avons calculé les fréquences pour les variables qualitatives. Nous avons calculé les moyennes, les médianes, l’écart type pour les variables quantitatives. Le test de Khi 2 ou le test de Fisher est utilisé pour comparer les variables qualitatives, le test de Student pour comparer les variables quantitatives. Le seuil de signification statistique a été fixé à 0,05.

## Résultats

**Figure 1:**
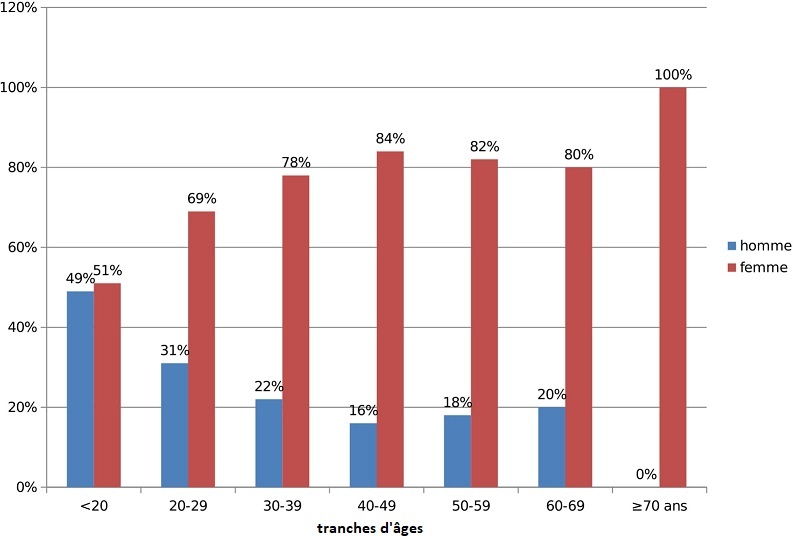
Répartition des patients en fonction du genre et des tranches d'âge

Notre étude a recensé 450 patients asthmatiques obèses constituant ainsi 21,4% des asthmatiques suivis ou hospitalisés durant la période de l’étude. L’âge moyen au moment du diagnostic était de 45±12,8 ans. La tranche d’âge entre 40 et 49 ans était prédominante avec un pourcentage de 30,4%. L’âge moyen de l’installation de l’asthme était de 38,7±12,5 ans. Le genre féminin était prédominant (81,3%). Cependant, la répartition en fonction de l’âge et du genre a montré une répartition égale entre les deux sexes avant l’âge de 20 ans (Figure 1). Le tabagisme était noté dans 3% des cas. L’IMC moyen était de 34,8±4,2 kg/m^2^ avec des extrêmes allant de 30 à 50 kg/m^2^. Plus que deux tiers des patients (61,3%) avaient une obésité grade I de l’OMS. Sur le plan clinique, le symptôme prédominant était la dyspnée sifflante avec une fréquence de 90.7%. Un asthme allergique a été retrouvé dans 51.3% des cas. Sur le plan fonctionnel, un déficit ventilatoire a été noté chez 34.2% des patients. L’asthme était bien contrôlé chez 55,3% des patients. Des critères de sévérité étaient trouvés dans 37,4% des cas. Onze pourcent des patients avaient un asthme difficile. Sur le plan thérapeutique, une corticothérapie inhalée a été prescrite chez tous nos patients. Selon GINA 2016, 26% des patients étaient traité par le palier 2, 48% selon le palier 3 et 24,2% selon le palier 4 (Tableau 1).

**Tableau 1:** Caractéristiques épidémiologiques et cliniques de la population étudiée

Variable	Tous les patients (n=450)	Groupe 1 (D<40) (n=236)	Groupe 2 (D≥40) (n=214)	Valeur de p
**Age (ans)**	45.5±12.8	38	53	-
**Sexe (féminin)**	81.3%	75.2%	87.5%	0.001
**IMC (kg/m2)**	34.8±4.2	34.9±5.6	34.7±5.6	0.3
**Atopie personnelle**	63%	71%	57.5%	0.003
**Allergie**	51.3%	62.5%	40.6%	<0.001
**Comorbidités ≥1**	41.3%	18.3%	47.1%	<0.001
**Déficit ventilatoire**	34.2%	32.6%	38.3%	0.242
**Sévérité de l'asthme**	37.4%	40.1%	35.3%	0.227
**Asthme difficile**	11%	10.1%	12.6%	0.354

n: nombre, IMC: indice de masse corporelle

Au terme de ce travail, nous avons distingué deux phénotypes de l’asthme associées à l’obésité et ceux-ci selon l’âge du début des symptômes. Le premier phénotype D1 (52.4%) était caractérisé par un asthme à début précoce, associé à une fréquence plus élevée d’allergie (62.5 versus 40.6%, p < 0,001), et des manifestations d'atopie (71 versus 57,5%, p = 0,003). Le deuxième phénotype (47,6%) était caractérisé par un asthme à début tardif, fréquemment associé au sexe féminin (87,5 versus 75,2%, p = 0,001) et un taux plus élevé de comorbidités (47.1 versus 18.3%, p<0.001) et d’hospitalisations (19.2 versus 11.3%, p=0.0014) (Tableau 1, Tableau 2). Par ailleurs, l’étude des paramètres de la sévérité de l’asthme selon les grades de l’obésité a montré que les obèses de grade II et III avaient un déficit ventilatoire important (capacité vitale forcée (CVF) moyenne = 1807 ml versus 2092 ml pour le grade I, et un VEMS moyen = 1421 ml versus 1680 chez le grade I, p= 0.0022 et 0.007 respectivement). Par ailleurs, une charge thérapeutique significativement plus importante (palier 3 ou 4) était notée chez ce groupe (Grade II et III: 79,3% versus grade I: 70,7%, p = 0,026).

**Tableau 2:** Tableau comparatif des caractéristiques évolutives entre les deux phénotypes.

Variable	Groupe 1 (D <40) (n=236)	Groupe 2 (D≥40) (n=214)	Valeur de p
Asthme sévère	36%	39.8%	0.294
Asthme partiellement/non contrôlé	42%	48.9%	0.093
Asthme difficile	10.1%	12.6%	0.354
hospitalisations	11.3%	19.2%	0.001

## Discussion

Ce travail avait pour objectif d'étudier les particularités du groupe asthmatiques obèses et de déterminer la relation entre les grades de l'obésité et la sévérité de la maladie. Nous avons pu définir deux phénotypes d'asthmatiques obèses: l'asthme à début précoce, associé plus fréquemment à l'allergie et à l'atopie, et l'asthme à début tardif, fréquemment associé au sexe féminin et un taux plus élevé de comorbidités et d’hospitalisations. D'autre part, l'obésité grade II et III était associée à un déficit ventilatoire plus important ainsi qu'une charge thérapeutique plus importante. Malgré la taille de notre population et la disponibilité de différentes données nécessaires à l'étude, notre travail n'est pas sans limites. En effet, vu la nature de l'étude rétrospective, nous n'avons pas d'idée sur la variabilité du contrôle de l'asthme en fonction du changement du poids chez un même patient. D'autre part, la prévalence exacte du syndrome d'apnée du sommeil (SAS) n'est pas appréciée dans notre population. En effet, outre l'obésité, le SAS est aussi un facteur de non contrôle de l'asthme. Enfin, l'observance thérapeutique ne peut pas être évaluée avec précision dans un travail rétrospectif.

La prévalence de l’obésité chez les asthmatiques était estimée dans notre étude à 21.4% avec une prédominance féminine. Les données épidémiologiques sur la fréquence des obèses chez les asthmatiques sont limitées. Selon une étude américaine en 2010, la fréquence de l’obésité chez les asthmatiques était de 38,8% [14]. Dans notre étude l’âge moyen au moment du diagnostic est concordant avec les données de la littérature ou la moyenne d’âge dans la majorité des études varie de 39 à 48.5 ans [15, 16]. Cependant, cette moyenne est supérieure à celle observée dans la population générale des asthmatiques [17, 18] étant donné que l’asthme de l’obèse est souvent d’installation tardive [19]. La prédominance du sexe féminin peut être expliquée par le taux d’obésité plus élevé dans la population féminine, le rôle des facteurs hormonaux particulièrement les œstrogène en favorisant le recrutement des éosinophiles, la libération de certaines cytokines de l’inflammation [20, 21], et le rôle de la leptine dont les concentrations sont plus importantes chez les obèses et particulièrement chez les femmes [22]. Une atopie personnelle a été noté chez 63,1% des patients ce qui est similaire aux données de la littérature [23, 24]. Néanmoins une étude australienne a montré que la présence de l’obésité ne semble pas influencer la fréquence de l’atopie [25]. Dans notre série, l'âge moyen de début des symptômes de 38.7±12.5 ans est similaire aux données de la littérature [25]. Sur le plan clinique, la dyspnée paroxystique sifflante augmente avec les classes de l’IMC représentant ainsi le symptôme le plus fréquent [26], tel est le cas dans notre étude.

Les méthodes d’analyse par regroupement «clusters» ont permis de mettre en évidence plusieurs phénotypes de l’asthme, dans lesquels l’âge du début et l’IMC sont des paramètres importants [27, 28]. Ainsi, dans la littérature et aussi dans notre étude, on a distingué deux phénotypes de l’asthme chez les obèses selon l’âge du début de la maladie [19, 29]. Le premier phénotype était caractérisé par un asthme à début précoce, associé à une fréquence plus élevée d’allergie avec une inflammation bronchique de nature éosinophilique. La surcharge pondérale constitue dans ce phénotype une complication de la maladie asthmatique [19,30]. Le deuxième phénotype était caractérisé par un asthme à début tardif, fréquemment associé au sexe féminin et un taux plus élevé de comorbidités et d’hospitalisations.

Les données de la littérature concernant la corrélation de la sévérité de l’asthme et l’obésité sont controversés. Nous n’avons pas trouvé une différence significative ce qui est similaire à plusieurs autres études [16, 31]. Cependant, certains auteurs ont trouvé une telle corrélation [15, 32, 33]. Il semble que la variabilité des résultats est due en partie à la diversité des critères de l’estimation de contrôle de l’asthme. Gibeon *et al*. [23], dans une étude incluant 666 cas, ont trouvé que l’asthme difficile était plus fréquent chez les obèses (IMC > 30 kg/m2) comparativement aux asthmatiques de poids normal. Cette fréquence élevée d’asthme difficile, peut être expliquée par la sévérité du déficit ventilatoire chez les sujets asthmatiques obèses, trouvés dans différentes études ainsi que chez les sujets de notre série. En effet, Sutherland *et al.* [34] (n = 1265), et Peters *et al.* [16] (n = 902) ont objectivé un déficit ventilatoire significativement important chez les asthmatiques obèses en comparaison aux asthmatiques en surpoids ou normaux.

Malgré l’importance de cette population d'asthmatiques obèses, les options thérapeutiques restent encore limitées avec un arsenal thérapeutique plus important (palier 3 ou 4) pour un meilleur contrôle [12, 35, 36]. Nos résultats rejoignent ceux rapportés par Boulet et Franssen [37], qui ont noté un besoin plus important en bronchodilatateur longue durée d’action associées à la corticothérapie inhalée (CI) chez les asthmatiques obèses de grade II et III en comparaison à leurs homologues de grade I. L’une des théories avancées dans la littérature pour expliquer ces constatations est la présence d’une faible sensibilité à la CI chez les asthmatiques obèses. A côté d’un traitement médical de l’asthme et de ses comorbidités, la perte de poids, une alimentation équilibrée ainsi que l’éducation et l’encadrement du patient semblent être nécessaire pour un meilleur contrôle de l’asthme et une meilleure qualité de vie [38, 39].

## Conclusion

L’obésité est l’un des facteurs clefs impliqués dans le mauvais contrôle de l’asthme. La prise en charge de l’asthme associée à l’obésité n’est pas encore codifiée. L’hétérogénéité de ce phénotype d’asthme impose une prise en charge multidisciplinaire basée essentiellement sur la valorisation de l’éducation du patient et la réduction de la surcharge pondérale en plus du traitement pharmacologique adopté. D'autres études prospectives sont nécessaires pour une meilleure compréhension de ce groupe d'asthmatiques obèses avec des implications thérapeutiques.

### Etat des connaissances actuelles sur le sujet

- L'asthme et l'obésité sont deux problèmes majeurs en santé publique avec des interactions mutuelles;
- L'asthmatique obèse représente un phénotype particulier de l'asthme avec des caractéristiques évolutives qui diffèrent d'une étude à une autre.

### Contribution de notre étude à la connaissance

- Le groupe des asthmatiques obèses est un groupe hétérogène;
- Nous avons distingué deux phénotypes dans ce groupe;
- Le déficit ventilatoire ainsi que la charge thérapeutique sont majorés dans les grades II et III de l'obésité.

## Conflits d’intérêts

Les auteurs ne déclarent aucun conflit d’intérêts.
